# Elevated external temperature affects cell ultrastructure and heat shock proteins (HSPs) in *Paramacrobiotus experimentalis* Kaczmarek, Mioduchowska, Poprawa, & Roszkowska, 2020

**DOI:** 10.1038/s41598-024-55295-z

**Published:** 2024-03-01

**Authors:** Pushpalata Kayastha, Filip Wieczorkiewicz, Myriam Pujol, Alison Robinson, Marek Michalak, Łukasz Kaczmarek, Izabela Poprawa

**Affiliations:** 1 Department of Animal Taxonomy and Ecology, Faculty of Biology, Adam Mickiewicz University in Poznań, Uniwersytetu Poznańskiego 6, 61-614 Poznań, Poland; 2 Institute of Biology, Biotechnology and Environmental Protection, Faculty of Natural Sciences, University of Silesia in Katowice, Bankowa 9, 40-007 Katowice, Poland; 3 Department of Biochemistry, Faculty of Medicine and Dentistry, University of Alberta, Edmonton, AB Canada

**Keywords:** Heat shock proteins, Storage cells, Ultrastructure, Tardigrades, Water bears, Ecology, Physiology, Structural biology, Environmental sciences

## Abstract

Increasing temperature influences the habitats of various organisms, including microscopic invertebrates. To gain insight into temperature-dependent changes in tardigrades, we isolated storage cells exposed to various temperatures and conducted biochemical and ultrastructural analysis in active and tun-state *Paramacrobiotus experimentalis* Kaczmarek, Mioduchowska, Poprawa, & Roszkowska, 2020. The abundance of heat shock proteins (HSPs) and ultrastructure of the storage cells were examined at different temperatures (20 °C, 30 °C, 35 °C, 37 °C, 40 °C, and 42 °C) in storage cells isolated from active specimens of *Pam. experimentalis*. In the active animals, upon increase in external temperature, we observed an increase in the levels of HSPs (HSP27, HSP60, and HSP70). Furthermore, the number of ultrastructural changes in storage cells increased with increasing temperature. Cellular organelles, such as mitochondria and the rough endoplasmic reticulum, gradually degenerated. At 42 °C, cell death occurred by necrosis. Apart from the higher electron density of the karyoplasm and the accumulation of electron-dense material in some mitochondria (at 42 °C), almost no changes were observed in the ultrastructure of tun storage cells exposed to different temperatures. We concluded that desiccated (tun-state) are resistant to high temperatures, but not active tardigrades (survival rates of tuns after 24 h of rehydration: 93.3% at 20 °C, 60.0% at 35 °C, 33.3% at 37 °C, 33.3% at 40 °C, and 20.0% at 42 °C).

## Introduction

The phylum Tardigrada currently consists of *ca*. 1,500 species (33 families, 159 genera, 1464 species, and 21 additional subspecies)^1^ that inhabit most terrestrial and aquatic environments worldwide^2–4^.

Tardigrades (water bears) are well-known extremophiles, organisms that are able to survive extreme conditions. Water is essential for tardigrade survival because it keeps them physiologically active. However, as they desiccate^5^ and form tuns, by constricting and retracting their head and legs to produce a barrel-shaped, quiescent body, their bodies lose roughly 95% of their water content^6–8^. To survive the extreme environmental conditions they experience, many species enter cryptobiosis, a state in which no metabolic activity occurs, preventing reproduction and development^9,10^. Cryptobiosis is a widespread and reversible state caused by, for example, desiccation, particularly in limno-terrestrial animals^11^. Different types of cryptobiosis are caused by various environmental stressors and include anhydrobiosis (caused by lack of water), anoxybiosis (lack of oxygen), chemobiosis^11^ (chemical composition), cryobiosis (low temperature), and osmobiosis (change in osmotic conditions)^9,12^. During this process various molecules may act as cytoprotectants, including specific proteins^13–16^. Currently, only a few proteins: Late Embryogenesis Abundant (LEA) proteins; Cytoplasmic Abundant Heat Soluble (CAHS) proteins; Secretory Abundant Heat Soluble (SAHS) proteins; Mitochondrial Abundant Heat Soluble (MAHS) proteins; Dsup (damage suppressor protein), as well as heat shock proteins, are recognized as cytoprotectants^17–21^. Various HSPs (heat shock proteins), including small heat shock proteins (sHSPs) and HSP70, are preserved in full range in tardigrades^22–24^. Studies of HSPs in tardigrades are primarily focused on their role in anhydrobiosis^22,25–27^. The sHSPs, such as HSP27 and HSP30C, are upregulated during dehydration in case of eutardigrade *Milnesium tardigradum*^28^, HSP20-3 and HSP20-6 are upregulated sHSPs in response to a 24 h heat shock at 35 °C of adult tardigrades in *Ramazzottius varieornatus*^29^; however, HSP70 is upregulated during recovery and rehydration^30–33^. Also, *Ram. varieornatus* showed nine heat shock related transcripts were upregulated (two HSP70 isoforms, one HSP90, one HSP68, one HSP60, and four small HSPs), whereas one HSP70 and two small HSPs where downregulated of various heat shock transcripts at 35 °C^34^. This suggests that the upregulation of small and large HSPs is a response to different stages of stress.

Higher temperature may damage organs, cells and cell components, *e.g.*, storage cells and organ systems such as the gut, cell membranes, mitochondria, DNA and proteins. Examples of such damage have been documented in various biological systems, such as sea cucumbers^35^, spermatid cells of rats^36^, and tobacco (*Nicotiana sylvestris*)^37^. Doyère^38^ and Pouchet^39^ conducted the first studies of tardigrade thermotolerance in the early nineteenth century, followed by Li and Wang^40^ who studied thermal adaptation of *Macrobiotus harmsworthi* (Murray, 1907^41^) and Rebecchi et al.^42^, who studied the tolerance of *Borealibius zetlandicus* (Murray, 1907^41^) at 25–37 °C for 1 h as well as after 24 h at 36 °C and 38 °C. In a more recent study, Neves et al.^43^ reported that *Ram. varieornatus* exhibited sensitivity to high temperature in active tardigrades and higher tolerance to high temperature in its anhydrobiotic states. Furthermore, heat stress tolerance of desiccated (anhydrobiotic) tardigrades appears to be limited by exposure time as the survival rate of *Ram. varieornatus* decreased when exposed to higher temperature for 24 h in comparison with the exposure for 1 h^44^. The latest study is by Giovannini et al. where the study shows that an Antarctic tardigrade species' (*Acutuncus antarcticus* (Richters, 1904^45^)) life history features and gene expression are changing as temperatures rise^46^.

Tardigrades lack a circulatory or respiratory system, but the body cavity is filled with storage cells, which float freely in the hemolymph or occasionally attach to the basement membranes of other tissues^47,48^. Storage cells are responsible for both nutrient transport and storage of lipids, polysaccharides, and pigments (such as carotenoids)^2,49^. They produce protein substances that combine to form lipid globules and vitellogenins in some species^2,50,51^. Storage cells are ameboid in shape, and their numbers and size are dependent upon the nutritional state of the animal^47,48^. Each storage cell has a very large lobular nucleus with a large, non-homogenous nucleolus in the center. Intracellular organelles, such as mitochondria and rough endoplasmic reticulum cisternae, are found in the cytoplasm. The cytoplasm also includes non-homogeneous spheres of various sizes^52^. Storage cells are easy to isolate; preserve the arrangement of their organelles. Storage cells also store and produce lipids, polysaccharides, and protein substances, making them ideal candidates for the study of heat-dependent structural and biochemical changes. Most importantly, both changes in storage cell ultrastructure and HSP expression as a function of increased temperature have not been reported in a single study of active and desiccated tardigrades. In the present study, we examined ultrastructural changes, as well as the abundance HSP27, HSP60, and HSP70, in storage cells isolated from heat-treated active specimens and anhydrobiotic tuns of *Paramacrobiotus experimentalis* Kaczmarek, Mioduchowska, Poprawa, & Roszkowska, 2020^53^. We selected to test the abundance of these specific HSPs after Schokraie et al.^27^ found many heat shock proteins, including the ones we employed in the eutardigrade species *Mil. tardigradum*. Furthermore, Hsp27 is increased after dehydration in cases of tardigrade. *Mil. tardigradum*, Hsp70 has already been reported as a molecular chaperone that may play a role in desiccation resistance, while hsp60 is an abundant protein and all of these HSPs play roles in anhydrobiosis.

## Materials and methods

### Animal model

*Paramacrobiotus experimentalis* specimens were extracted from a sample of mosses collected near Fort-Voyron, Antananarivo, Antananarivo Province, Madagascar (18° 55′ 35″ S, 47° 31′ 23″ E, 1,340 m.a.s.l.). Fully active *Pam. experimentalis* specimens were used to establish a laboratory population in culture medium (spring water (Żywiec Zdrój): double distilled water (ddH_2_O) in a 1:3 ratio) on scratched Petri dishes, following the protocol of Roszkowska et al.^54^. Rotifers (*Lecane inermis*) and nematodes (*Caenorhabditis elegans*) were added ad libitum as a food source once per week. Adult, fully active, and non-molting *Pam. experimentalis* specimens of a medium body size (~ 450 μm) were selected for experiments.

### Experimental design

#### Heat stress protocol for active animals

All experiments were performed in 1.5 mL Eppendorf tubes containing 10 specimens in 1 mL culture medium. Three to five independent experiments were conducted for each experimental condition. Each of the Eppendorf tubes was then placed on a heat block (Benchmark digital heat block, BSH1002) with an open lid for 5 h at a different temperature: 20 °C, 35 °C, 37 °C, 40 °C, and 42 °C. Thereafter, the specimens were used for ultrastructural and biochemical analysis. These timepoint and temperatures were chosen as experimental groups based on thermotolerance experiments known from literature and performed on various tardigrades species (for e.g., Refs.^43,55^).

#### Anhydrobiosis protocol

All experiments were performed in covered, vented plastic Petri dishes (Ø 35 mm) lined on the bottom with white filter paper (grammage 85–87, Chemland Company, Poland) (n = 15). The specimens were transferred into dishes, along with 450 µL of the culture medium, using an automatic pipette. Then, the dishes were placed into a climate chamber (PolLab, Q-Cell 140), and the individuals were allowed to dry slowly in the dark for 72 h at 20 °C, with 40–50% relative humidity^56–60^. Once every 24 h, we monitored for tun formation using a stereomicroscope. Once tuns were formed, the specimens were subjected to 20 °C, 35 °C, 37 °C, 40 °C, and 42 °C for 5 h. Then, half of the tuns were directly fixed for light and transmission electron microscopy, and the other half were left overnight to rehydrate in culture medium for anhydrobiosis success analysis. To check the anhydrobiosis success rate, specimens (n = 15) were rehydrated for 24 h using the culture medium. Thereafter, active animals were counted, and the percentage of active tardigrades was calculated. These timepoint and temperatures were chosen as experimental groups based on thermotolerance experiments from literature performed on various tardigrades species.

### Light and transmission electron microscopy

Active or tun-state *Pam. experimentalis* (*n* = 15 for each condition) were subjected to 20 °C, 35 °C, 37 °C, 40 °C, or 42 °C for 5 h. Then, the material was fixed with 2.5% glutaraldehyde, postfixed in 2% osmium tetroxide, dehydrated, and embedded according to the protocol described by Janelt et al.^61^. Semi-thin (800 nm) and ultra-thin (70 nm) sections were cut using a Leica Ultracut EM UC7 ultramicrotome. Semi-thin sections were stained with 1% methylene blue in 1% borax and analyzed using an Olympus BX60 light microscope. Ultra-thin sections were mounted on copper grids and stained with uranyl acetate and lead citrate. Material was examined using a Hitachi H500 transmission electron microscope at 75 kV or a Hitachi UHR FE-SEM SU 8010 scanning electron microscope equipped with an ET (Everhart–Thornley) detector for imaging the sections at a low voltage of 25 kV.

### Flow cytometry analysis of HSPs

For each condition, 15 active specimens were collected into 1.5 mL Eppendorf tubes in a total of 500 µL of water and exposed to 20 °C, 35 °C or 42 °C for 5 h. Next, the active state specimens (both alive and dead after heat treatment) were transferred to a single depression concave slide with phosphate-buffered saline (PBS) and dissected using a BD precision needle (27 G × ½ in.) (Becton Dickinson). The storage cells were carefully collected and transferred to 1.5 mL Eppendorf tubes in a total volume of 150 µL of PBS. Cells were fixed with a fixation/permeabilization buffer (eBioscience Catalog number: 88-8824-00) for 30 min at room temperature (RT), washed with a blocking buffer containing 1X permeabilization buffer (eBioscience Catalog number: 00-8333-56) with 1% BSA (Bovine serum albumin), and centrifuged at 1500×*g* for 5 min. Cells were incubated for 40 min at RT with 20 µL of anti-HSP27 (Ms anti-HSP27 Stress Marq SMC-161b), anti-HSP60 (HSP60 (D307) Antibody, Cell Signaling Technology), or anti-HSP70 (Rab anti-HSP70 Stress Marq SPC-103D; 1:25 dilution) in the blocking buffer (*i.e.*, permeabilization buffer (eBioscience) with 1% BSA). Cells were washed with the permeabilization buffer and 10 µL of diluted 488 donkey anti-mouse antibodies (Invitrogen) or Alexa Fluor 647 goat anti-rabbit antibodies (Invitrogen) (1:120 final dilution in blocking buffer) were added to each tube, incubated for 1 h, and then washed in blocking buffer by centrifugation at 1500×*g* for 5 min. The basal fluorescence levels were defined by using a sample labelled only with secondary antibodies. Single events were recorded using an LSRFortessa™ X-20 flow cytometer (BD Biosciences Pharmingen). Data analysis was performed using FlowJo™ v10 for PC (TreeStar). Three to five independent experiments were conducted for each experimental condition.

### Statistical analysis for HSP data

The mean fluorescent intensity observed was normalized using control (20 °C) data. We analyzed the differences between treatments using a nonparametric Friedman test. A value of *p* < 0.05 was deemed statistically significant.

### Ethical approval

Samples of moss/lichen containing *Pam. experimentalis* were collected according to research permission from the Direction Generale des Forests, Direction de la Valorisation des Ressources Forestieres, Antananarivo, Madagascar (autorisations de recherche: No: 260/15-MEEMF/SG/DGF/DCAP/SCBT and Service de la Gestion Faune et Flore No: 056N-EA03/MG18). The research does not involve species at risk of extinction.

## Results

### Ultrastructural analysis of active specimens

At 35 °C, active individuals of *Pam. experimentalis* shrunk, tucking their legs and head, but did not form tuns, as in anhydrobiosis. After being transferred to RT, they resumed activity. However, active individuals incubated at 37 °C, 40 °C, or 42 °C had firmly erect bodies, and after being transferred to RT, they did not resume activity. The survival rates of active animals after 5 h at 20 °C, 35 °C, 37 °C, 40 °C and 42 °C and wait of 3 h were as follows: 100.0% at 20 °C, 100.0% at 35 °C, 0.0% at 37 °C, 0.0% at 40 °C, and 0.0% at 42 °C.

**Figure 1:**
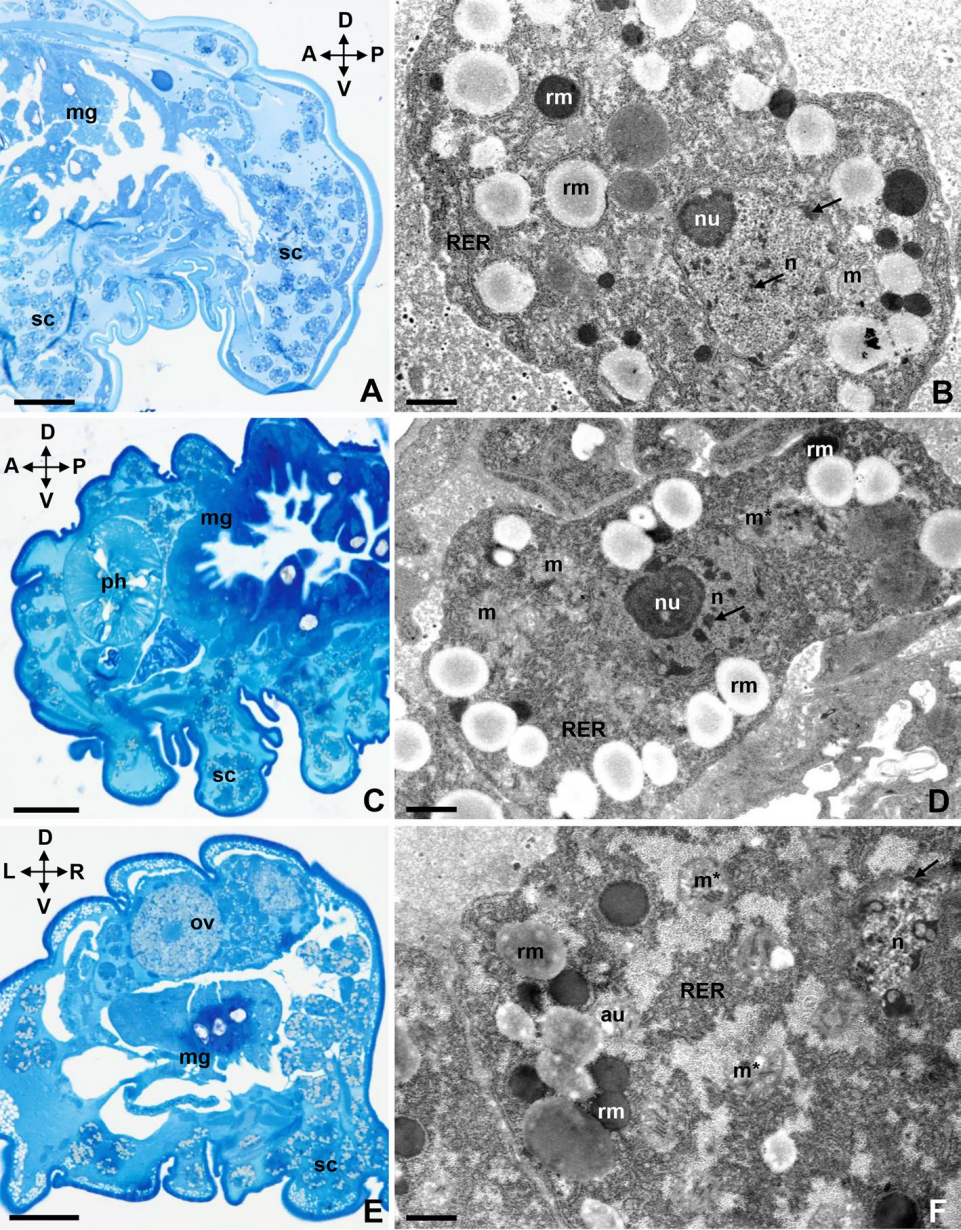
The storage cells of the active specimens of *Paramacrobiotus experimentalis* incubated under different temperature conditions (20 °C/35 °C/37 °C). (**A**) The storage cells of a control animal (20 °C), longitudinal section, LM, scale bar 20 µm, (**B**) the ultrastructure of a storage cell of a control animal (20 °C), TEM, scale bar 0.68 µm, (**C**) the storage cells of an animal exposed to 35 °C, longitudinal section, LM, scale bar 20 µm, (**D**) the ultrastructure of a storage cell of an animal exposed to 35 °C, TEM, scale bar 0.68 µm. (**E**) The storage cells of an animal exposed to 37 °C, transverse section, LM, scale bar 20 µm, (**F**) the ultrastructure of a storage cell of an animal exposed to 37 °C, TEM, scale bar 0.68 µm. Note that: A ↔ P indicates the anteroposterior axis, D ↔ V is the dorsoventral axis, while L ↔ R denote the left and right sides. *au* autophagic structure, *arrow* heterochromatin, *mg* midgut, *m* mitochondrion, m* damaged mitochondrion, *n* nucleus, *nu* nucleolus, *ov* ovary, *ph* pharynx, *rm* reserve material, *RER* rough endoplasmic reticulum, *sc* storage cells.

At 20 °C, isolated storage cells of the control group remained in the body cavity among the internal organs (Fig. 1A, sc). The cells had amoeboid or spherical shapes (Fig. 1A,B). A lobular nucleus with a heterogeneous nucleolus occupied the central part of each cell. The internal region of the nucleolus had a lower electron density than the external region. The nucleolus was surrounded by clumps of heterochromatin (Fig. 1B). The cytoplasm was rich in cisternae of the rough endoplasmic reticulum, mitochondria with visible cristae and low electron density of the matrix, ribosomes, and spheres of the reserve material (Fig. 1B). These spheres varied in size and electron density.

Similar to the storage cells isolated from control specimens, storage cells isolated from individuals subjected to 35 °C for 5 h had amoeboid or spherical shapes (Fig. 1C,D). Additionally, each cell’s nucleus had a prominent heterogeneous nucleolus that occupied the central part of the cell (Fig. 1D). However, we observed changes in mitochondria morphology in cells from specimen exposed to 35 °C. Several mitochondria degenerated, gradually losing cristae. Their matrices had higher electron densities than those of undamaged mitochondria (Fig. 1D).

The storage cells isolated from the specimens subjected to 37 °C for 5 h retained amoeboid or spherical shapes (Fig. 1E,F), but some of the ultrastructural features were altered. There were numerous degenerating mitochondria, which showed a loss of cristae and a matrix with higher electron density, and autophagic structures in the cell cytoplasm (Fig. 1F). The cisternae of the rough endoplasmic reticulum exhibited fragmentation. Moreover, we observed areas of the cytoplasm with low electron density (Fig. 1F). The nucleus and the reserve material sphere in the cytoplasm showed no visible changes.

**Figure 2:**
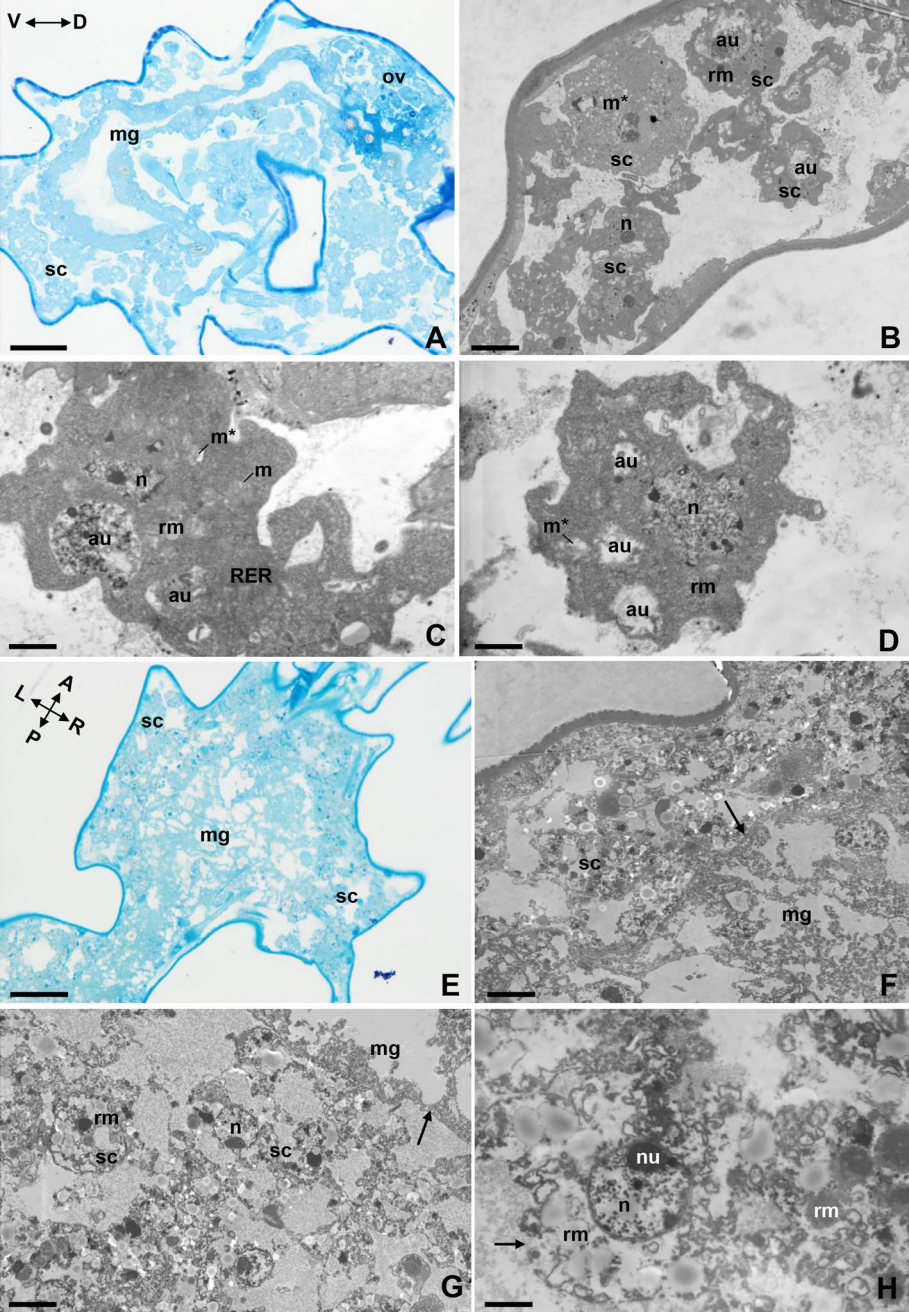
The storage cells of active specimens of *Paramacrobiotus experimentalis* incubated under different temperature conditions (40 °C/42 °C). (**A**) The storage cells of an animal exposed to 40 °C, transverse section, LM, scale bar 20 µm. (**B**–**D**) The ultrastructure of storage cells of animals exposed to 40 °C, TEM, (**B**) scale bar 4 µm, **C.** scale bar 1.1 µm, (**D**) scale bar 1.25 µm, (**E**) the storage cells of an animal exposed to 42 °C, longitudinal section, LM, scale bar 20 µm, (**F**–**H**) the ultrastructure of storage cells of animals exposed to 42 °C, TEM, **F.** Basal lamina (arrow), scale bar 4.2 µm. (**G**) Basal lamina (arrow), scale bar 4 µm, (**H**) damaged cell membrane (arrow), scale bar 1.2 µm. Note that: A ↔ P indicates the anteroposterior axis, D ↔ V is the dorsoventral axis, while L ↔ R denote the left and right sides. *au* autophagic structure, *mg* midgut, *m* mitochondrion, m* damaged mitochondrion, *n* nucleus, *nu* nucleolus, *ov* ovary, *rm* reserve material, *RER* rough endoplasmic reticulum, *sc* storage cells.

The shapes of the storage cells of the specimens subjected to 40 °C for 5 h were highly irregular (Fig. 2A–D). Many storage cells were shrunk, with many degenerated mitochondria with lost cristae. The mitochondrial matrix showed medium electron density or was electron lucent (Fig. 2C,D). Numerous autophagic structures were present in the cytoplasm of the cells (Fig. 2B–D). No changes were observed in cell nuclei or spheres of the reserve material (Fig. 2B–D).

The most extensive changes were observed in storage cells isolated from active individuals exposed to 42 °C (Fig. 2E–H). In these animals, cells and all organs degenerated and were difficult to distinguish (Fig. 2F–H). The midgut was identifiable only in that it retained the basal lamina (Fig. 2F,G). The storage cells had spherical shapes (Fig. 2G) and showed features typical of necrosis. The cytoplasm became electron lucent, and the cell membranes ruptured (Fig. 2H), releasing the cell contents into the body cavity (Fig. 2F–H). The cell nucleus had distended and assumed a spherical shape, but the nucleolus was still visible (Fig. 2G,H). Mitochondria and the cisternae of the rough endoplasmic reticulum were no longer evident, while spheres of the reserve material were still visible (Fig. 2H).

### Ultrastructural analysis of desiccated specimens (tun stage)

**Figure 3:**
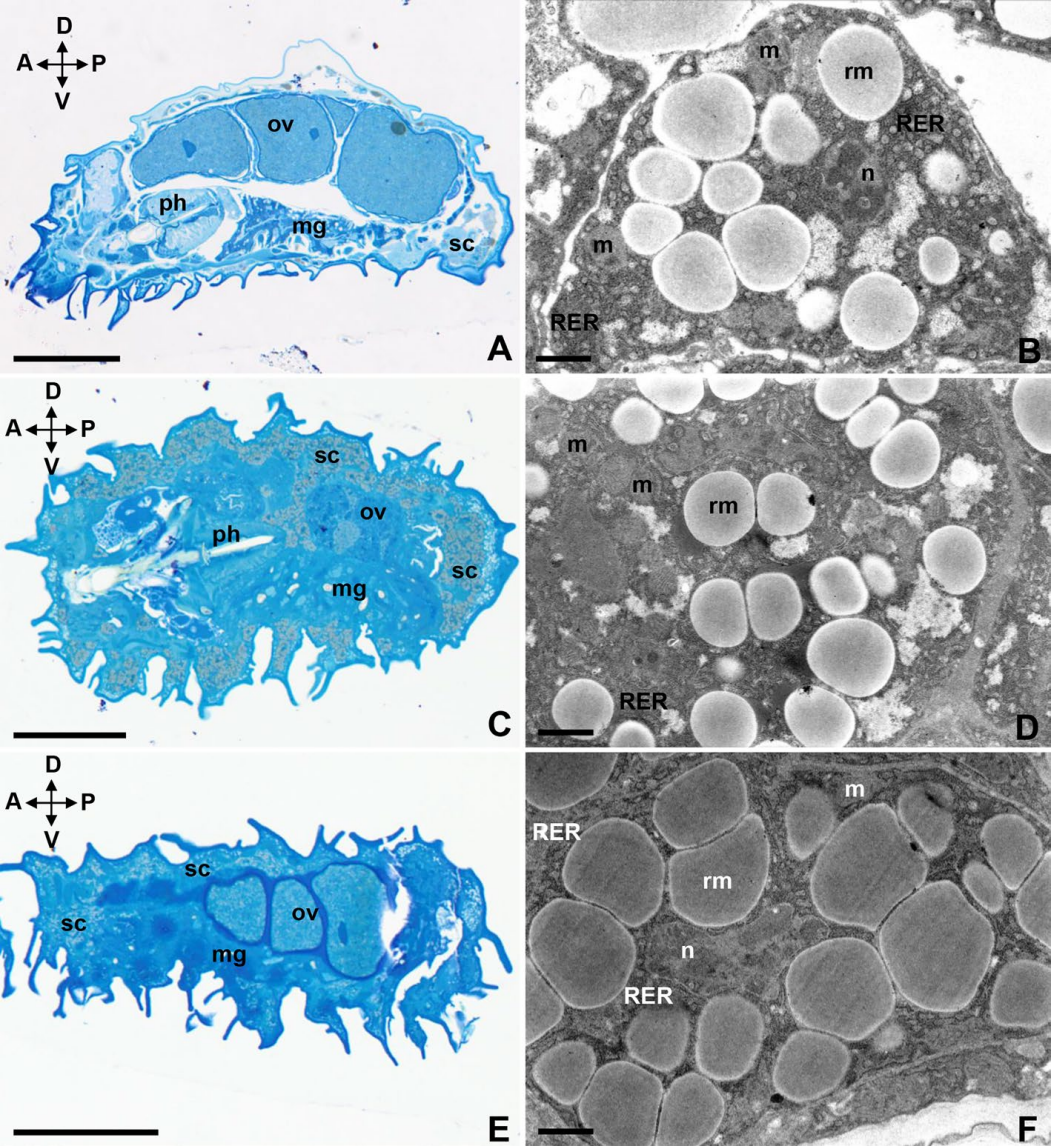
The storage cells of tuns of *Paramacrobiotus experimentalis* incubated under different temperature conditions (20 °C/35 °C/37 °C). (**A**) The storage cells of a tun-state control animal (20 °C), longitudinal section, LM, scale bar 50 µm, (**B**) the ultrastructure of a storage cell of a tun-state control animal (20 °C), TEM, scale bar 0.6 µm, (**C**) the storage cells of a tun exposed to 35 °C, longitudinal section, LM, scale bar 50 µm, (**D**) The ultrastructure of a storage cell of a tun exposed to 35 °C, TEM, scale bar 0.6 µm, (**E**) the storage cells of a tun exposed to 37 °C, longitudinal section, LM, scale bar 50 µm, (**F**) the ultrastructure of a storage cell of a tun exposed to 37 °C, TEM, scale bar 0.6 µm. Note that: A ↔ P indicates the anteroposterior axis while D ↔ V is the dorsoventral axis. *mg* midgut, *m* mitochondrion, *n* nucleus, *ov* ovary, *ph* pharynx, *rm* reserve material, *RER* rough endoplasmic reticulum, *sc* storage cells.

**Figure 4:**
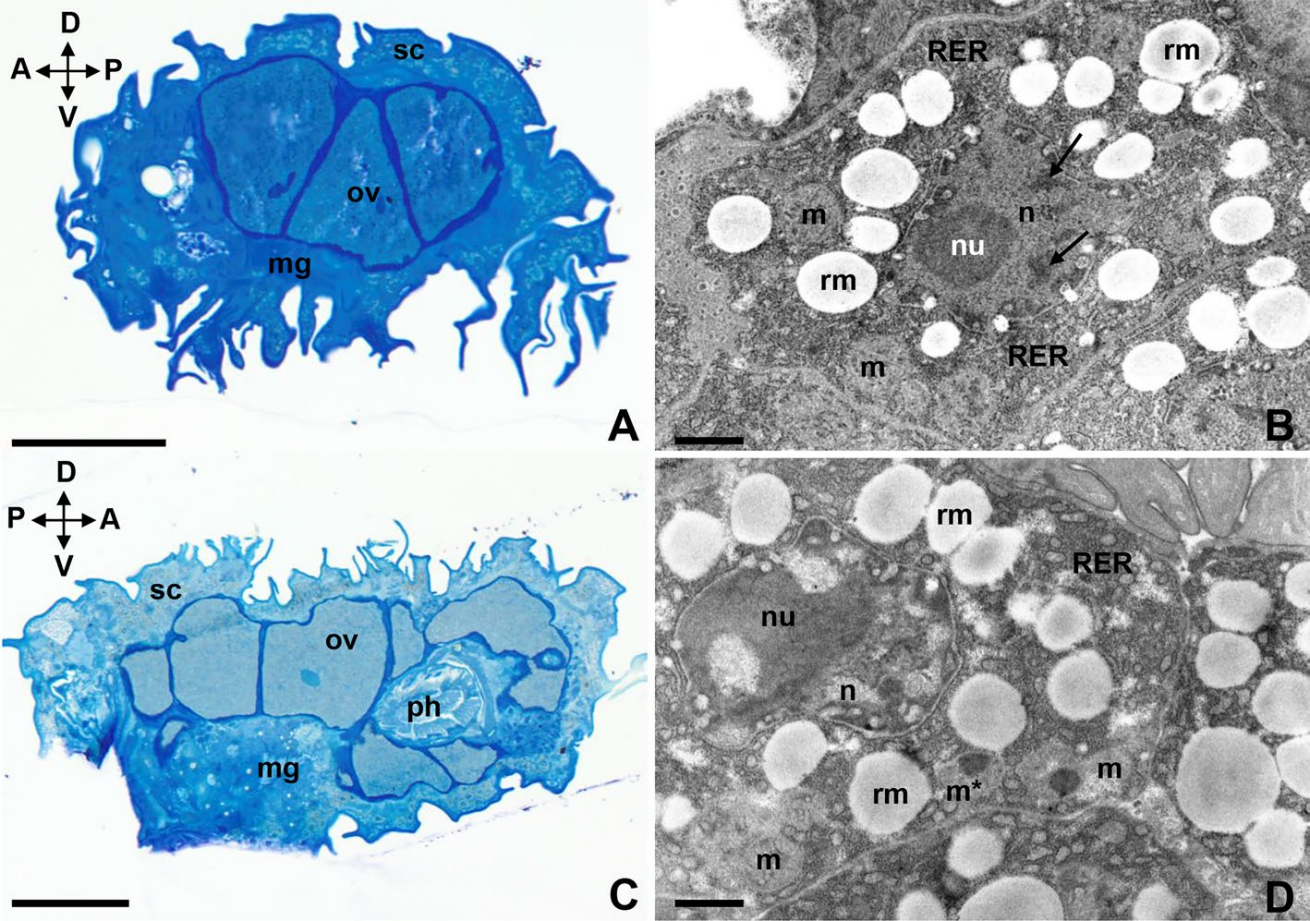
The storage cells of tuns of *Paramacrobiotus experimentalis* incubated under different temperature conditions (40 °C/42 °C). (**A**) The storage cells of a tun exposed to 40 °C, longitudinal section, LM, scale bar 50 µm, (**B**) the ultrastructure of a storage cell of a tun exposed to 40 °C, TEM, scale bar 0.8 µm, (**C**) the storage cells of a tun exposed to 42 °C, longitudinal section, LM, scale bar 50 µm, (**D**) the ultrastructure of a storage cell of a tun exposed to 42 °C, TEM, scale bar 0.8 µm. Note that: A ↔ P indicates the anteroposterior axis while D ↔ V is the dorsoventral axis. *arrow* heterochromatin, *mg* midgut, *m* mitochondrion, m* damaged mitochondrion, *n* nucleus, *nu* nucleolus, *ov* ovary, *ph* pharynx, *rm* reserve material, *RER* rough endoplasmic reticulum, *sc* storage cells.

The survival rates of tuns after 24 h of rehydration were as follows: 93.3% at 20 °C, 60.0% at 35 °C, 33.3% at 37 °C, 33.3% at 40 °C, and 20.0% at 42 °C. Storage cells occupied all free spaces in each tun's body cavity. The analysis of the ultrastructure of the storage cells of tuns subjected to the experimental temperatures (35 °C, 37 °C, 40 °C, and 42 °C) showed no differences compared to cells isolated from control tuns (20 °C) (Figs. 3A–F, 4A–D). These storage cells had shrunk and had amoeboid shapes. The cytoplasm had a high electron density (Figs. 3F, 4B). Frequently, lower electron density was observed in the cytoplasm (Figs. 3B,D, 4D). In the central part of the cell, there was an irregularly shaped nucleus (Figs. 3B,F, 4B,D) with a nucleolus (Fig. 4B,D). The nucleolus was surrounded by clumps of heterochromatin-like structures (Fig. 4B). In the storage cells of tuns exposed to 42 °C, karyolymph had a higher electron density, and large nucleolar vacuoles were visible (Fig. 4D). In the mitochondria, due to the density of the matrix, the cristae were hardly visible (Figs. 3B,D,F, 4B,D). Electron-dense material had accumulated in the few remaining mitochondria of storage cells exposed to 42 °C (Fig. 4D). In the cytoplasm of the storage cells of both the control group and all experimental groups, distended cisternae of the rough endoplasmic reticulum and spheres of reserve material were visible. These spheres varied in size and had medium electron densities (Figs. 3B,D,F, 4B,D).

### Abundance of HSPs in storage cells of active animals (preliminary findings)

**Figure 5:**
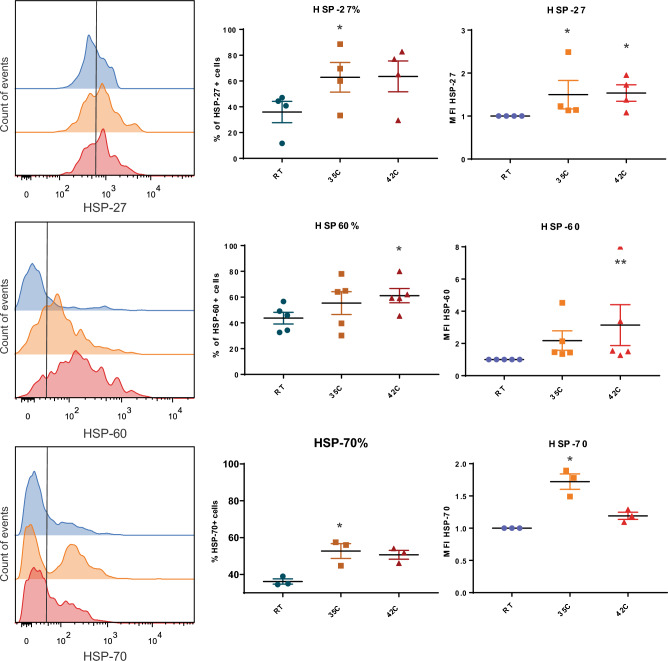
Levels of heat shock proteins (HSPs) in *Pam*. *experimentalis*. (**A**) Representative histograms of the flow cytometry analysis of HSP27, HSP60, and HSP70 in storage cells from tardigrades incubated at 20 °C, 35 °C, and 42 °C. The y axis shows the count of events for gated storage cells (according to forward scatter (FSC) and side scatter (SSC) parameters) and the x axis shows the intensity of fluorescence for each HSP marker. The negative fluorescence was determined by using secondary antibody control; (**B**) percentage of HSP27^+^, HSP60^+^, and HSP70^+^ storage cells from tardigrades at 20 °C, 35 °C, and 42 °C; **C.** Mean fluorescence intensity (MFI) values for HSP27^+^, HSP60^+^, and HSP70^+^ storage cells from tardigrades at 20 °C, 35 °C, and 42 °C. Levels of significance are represented as: *p ≤ 0.05, **p ≤ 0.01. Data are from three to five independent experiments.

The changes in the abundance of HSPs were examined using flow cytometry in active animals at three different temperatures: 20 °C (control group, optimal temperature for tardigrade survival), 35 °C (highest temperature at which active animals survived), and 42 °C (all animals died). The histograms (Fig. 5A) show the detected fluorescence for the different HSPs. The graphs show the percentages of HSP^+^ cells (Fig. 5B), and the mean fluorescent intensity (MFI) values (Fig. 5C) corresponding only to the HSP^+^ cell population. Approximately, 36.0% of the storage cells were positive for HSP27 when tardigrades were exposed to 20 °C, while the percentages of HSP27^+^ cells increased to 62.9% (p = 0.0133) in animals exposed to 35 °C or 63.6% (p = 0.0771, ns) when exposed to 42 °C (Fig. 5B). The MFI values for HSP27^+^ cells showed a similar increase after exposure to 35 °C or 42 °C (1.5 fold increase p = 0.0339) (Fig. 5C). The percentages of HSP60^+^ cells were also increased after exposure to 35 °C (55.3% p = 0.1138, ns) or 42 °C (61.2% p = 0.0269) compared to 20 °C (43.7%) (Fig. 5B). The MFI values were also increased at 35 °C (2.2 fold increase p = 0.578, ns) or 42 °C (3.1 fold increase p = 0.0044) (Fig. 5C). The analysis of HSP70 showed 36.2% of HSP70^+^ cells at 20 °C and these numbers increase to 52.7% (p = 0.0412) at 35 °C or 50.1% (p = 0.1025, ns) at 42 °C (Fig. 5B). The MFI values were increased by 1.7 fold (p = 0.0143) at to 35 °C and by 1.2 fold (p = 0.2207, ns) at 42 °C (Fig. 5C). So, there was significant increase in MFI values for HSP27 at both 35 °C and 42 °C, HSP60 at 42 °C and HSP70 at 35 °C. We concluded that there was a parallel increase in the abundance of all three heat shock proteins in animals exposed to increased external temperatures, compared to controls.

## Discussion

Although tardigrades are known for their ability to survive highly unfavorable environmental conditions in a state of anhydrobiosis, relatively little is known about their resistance to heat stress^10–12,50^, especially in active individuals^43^.

In the present study, we compared temperature-induced ultrastructural changes (at 20 °C, 35 °C, 37 °C, 40 °C, and 42 °C) in both active *Pam*. *experimentalis* and those in anhydrobiosis. Active specimens were susceptible to elevated temperatures. No individual survived 5 h at 37 °C, 40 °C, or 42 °C. This species was much more sensitive than *Ram. varieornatus*, which exhibited 50% mortality at 37.1 °C^43^.

Storage cells typically float freely in the fluid in the body cavity of tardigrades, but they occasionally attach to the internal organs. Storage cells contain reserve material (lipids, polysaccharides, and proteins) and pigments^2,47,48,50,51,62–64^. The reserve material is an energy reservoir that allows tardigrades to survive unfavorable environmental conditions, including entering a state of anhydrobiosis and returning to an active state^25,47,50–52,63,65,66^.

In the species studied, initial changes in the ultrastructure of storage cells were observed in the active individuals subjected to 35 °C. In these individuals, degeneration of few mitochondria was evident by loss of their cristae. Mitochondria play an important role in cell physiology, aging, and cell death; therefore, they are among the first organelles to be affected by changes in environmental conditions^67^. Mitochondrial changes similar to those we observed in *Pam*. *experimentalis* have also been described for *Hypsibius exemplaris*^68^ individuals incubated in paracetamol for 7 days at a concentration of 1 mg/L and for 28 days at concentrations of 0.2 μg/L, 230 μg/L, and 1 mg/L^64^. Although this was due to chemical stress, the result was similar to that induced by heat stress in our experiment. Loss of mitochondrial cristae has also been observed in somatic and germline cells of various invertebrates exposed to toxic metals^67,69,70^ and in the midgut cells of starved shrimp (*Neocaridina davidi*)^71^. Because mitochondria play a crucial role in the activation of cell death^72^, changes in both mitochondrial ultrastructure and mitochondrial membrane potential are expected to activate cell death^71,73–76^. Here, we showed that increased environmental temperature also has profound effects on mitochondrial architecture.

In the storage cells of active *Pam*. *experimentalis* incubated at 37 °C, mitochondria degenerated, exhibiting lost cristae in addition to numerous autophagic structures. More striking changes occurred in the storage cells of individuals incubated at 40 °C. Autophagy is the process during which damaged organelles, such as mitochondria, cisternae of the endoplasmic reticulum, or structures accumulating xenobiotics, are neutralized inside autophagosomes. Autophagy is aimed at protecting the cell from death^70,77–79^. An increase in autophagic structures in tardigrade storage cells incubated at higher temperatures indicated activation of the cells’ defense mechanisms. Increased autophagy has also been observed in the storage cells of *Hys*. *exemplaris* treated with paracetamol^64^ and in midgut cells of *Grevenius granulifer* infected with microsporidia^80^. In the former case, autophagy was responsible for eliminating damaged cell organelles, and, in the latter, for removing parasites. Accumulating too many autophagic structures in the cell activates the cell death pathway. Here, we showed that dramatic changes in storage cells, caused by high temperature, induced autophagy.

Exposure to 42 °C caused the most damage to active individuals. The cytoplasm of *Pam*. *experimentalis* storage cells became electron lucent, the cell membranes broke, and the degraded cells and organs displayed classic symptoms of necrosis. Necrosis is a type of cell death that can be induced by various stressors, such as external factors, mechanical trauma, or even intensive autophagy^76,81,82^. The interaction between autophagy and necrosis has been documented in the midgut cells of tardigrades^82^ and the fat bodies of myriapods^83^. Again, we showed that high temperature induced these changes.

Active individuals incubated at a higher temperature (37 °C, 40 °C, or 42 °C) did not survive the experiment. An approximate lifespan under experimental conditions can be estimated by considering the ultrastructural changes in their storage cells. Specimens incubated at 37 °C showed relatively minor structural change, so they probably died toward the end of the 5 h experiment. By contrast, specimens incubated at 42 °C died at the beginning of the experiment, as evidenced by the advanced necrosis of their internal organs and storage cells.

As in previous work on *Richtersius coronifer*^52^, the storage cells of anhydrobiotic specimens of *Pam*. *experimentalis* were smaller than those of active individuals and had an amoeboid shape. Due to cell shrinkage caused by water loss, the cytoplasm was electron dense. Similar changes were observed in the storage cells of anhydrobiotic tuns of *Pam*. *experimentalis* in the present study.

Ultrastructural analysis of storage cells in tuns of *Pam*. *experimentalis* showed no differences between the cells exposed to certain elevated temperatures (35 °C, 37° C, or 40 °C) and a control group (20 °C). However, in the tun storage cells incubated at 42 °C, the karyolymph became denser, and electron-dense material accumulated in the mitochondria. Czerneková et al.^52^, in their study on the effect of temperature on 6-month-old tuns of *Ric*. *coronifer*, also found no ultrastructural changes in storage cells of tuns incubated for 24 h at 50 °C. They reported that heating the *Ric*. *coronifer* tuns reduced survival significantly. Additionally, animals subjected to more intense heating generally required more time to rehydrate before being revived^52^. Interestingly, Ramløv and Westh^84^, who analyzed the effect of temperature on tuns of *Ric*. *coronifer*, found no effect on animal survival at 50–70 °C. By contrast, survival fell to 20% at 80 °C and 0% at 100 °C. It is difficult to explain these discrepancies. It is likely that, in the study by Czernekova et al.^52^, the 6 months of desiccation prior to exposure to 50 °C may have rendered the specimens more susceptible to heat stress. Because repair mechanisms are not operating during anhydrobiosis, damage from oxidative interactions with the surrounding air accumulates over time^42^. The non-heated animals did not show reduced survival after 6 months. The reason for this difference might be that the body may have been more sensitive to heat injury or less able to mend the combined damage from long-term desiccation and heating. It appears that the chemical elements required for cell survival, rather than overall cell architecture, were damaged, causing these harmful effects. In the present study, survival fell to 60.0% at 35 °C, 33.3% at both 37 °C and 40 °C, and 20.0% at 42 °C. At temperatures between 42 and 45 °C, protein denaturation occurs in cellular organelles^85–87^, which might be another reason for reduced survivability at higher temperatures. Since the durations of both anhydrobiosis and higher temperature exposure in the present study were minimal, changes in the ultrastructure of the storage cells were unlikely to significantly affect animal survival. Additional events, including damage to proteins and other chemical compounds, may play a significant role in the proper functioning of the cell. This is supported by our analysis of the expression of HSPs in heat-treated organisms *Pam*. *experimentalis*.

Three different HSPs (HSP27, HSP60, and HSP70) in active specimens of *Pam*. *experimentalis* at 20 °C, 35 °C, and 42 °C were analyzed using flow cytometry.

Even in cells that are not under stress, HSPs and their molecular partners have been shown to play a variety of roles in the folding, assembly, intracellular localization, secretion, regulation, and destruction of other proteins^88,89^. However, for cytoprotection from extreme cellular stress, including increased temperature, the rapid expression of inducible HSP70 is essential. Due to their rapid and substantial upregulation in response to a variety of environmental stressors, proteins in the HSP70 family frequently have been employed as biomarkers^90^. The HSP70 expression appears to be elevated in active specimens following the induction of heat stress. Also, HSP70 chaperones assist the refolding of heat-denatured peptides to reduce proteolytic destruction, a conserved process in eukaryotes known as the heat shock response^91^. Several heat-soluble protein families, also called the "tardigrade-unique proteins", as well as the highly conserved heat-inducible HSP70 family, are among the proteins suspected to play a role in tardigrade thermotolerance. They function as molecular barriers during anhydrobiosis^17,19,92^. Their function may be related to mechanisms of repair following stress, such as desiccation^31^.

Likewise, under heat stress, the mitochondrial HSP60 has been shown to form complexes with a number of polypeptides. The HSP60 is a well-known chaperone and is stress inducible^93^. Also, HSP60 is necessary to stop native dihydrofolate reductase (DHFR) imported into mitochondria from becoming thermally inactive in vivo. When DHFR was being thermally denatured in vitro, HSP60 linked to it, preventing it from aggregating and mediating its adenosine triphosphate–dependent refolding at higher temperatures. This is a generic mechanism by which proteins in the HSP60 family stabilize pre-existing proteins under stressful circumstances^94,95^. Furthermore, corrigendum to Neves et al.^96^ reported nine heat shock-related transcripts were increased (two HSP70 isoforms, one HSP90, one HSP68, one HSP60, and four small HSPs), with one HSP70 and two small HSPs being downregulated. Eleven CAHS, twelve SAHS, one MAHS, one RvLEAM, and one Dsup showed differential expressions. Two CAHS and three SAHS transcripts were elevated, while the remaining eutardigrade-specific transcripts were down-regulated. The HSP60 isoform, not RvLEAM or MAHS, may protect *Ram. varieornatus* mitochondria from heat stress. In the present study, the expression of HSP60 was also upregulated with heat stress. Because of their constant exposure to temperature fluctuations, cells increase their resistance to stress by upregulating the expression of HSP60 when necessary^93^.

Nonetheless, HSP27, a sHSP, also showed increased expression at higher temperatures. In both prokaryotic and eukaryotic cells, sHSPs were identified as a collection of proteins with a molecular mass ranging from 15 to 42 kDa^97^. Through their involvement in the modulation of cellular redox states, sHSPs were recognized to play a role in the regulation of other cellular functions, such as apoptosis and differentiation, in mammalian cells, in addition to promoting cell survival in response to stress^98^. Furthermore, two crystallin sHSPs (Mt-sHSP17.2 and Mt-sHSP19.5) from the tardigrade *Mil. inceptum*^99^ were reported to form sizable complexes under heat stress conditions, potentially stabilizing the structure of other proteins^25^. Interestingly, despite not being regulated during anhydrobiosis, the expression of one of these HSPs (Mt-sHSP17.2) is elevated by heat shock treatment of active specimens^25^. Recently, Hibshman et al.^100^ discovered that sHSPs were among the most abundant transcripts recorded in two separate RNA-seq datasets of *Hys. exemplaris*, which implies that sHSPs may be more transcriptionally responsive to temperature changes than large HSPs. Also, Al-Ansari et al.^29^ identified two members of the small heat shock protein (sHSP) family in *Ram. varieornatus*, HSP20-3 and HSP20-6. These are the most strongly upregulated sHSPs in adult tardigrades after a 24-h heat shock at 35 °C, with HSP20-3 being one of the most significantly elevated across the transcriptome.

## Data Availability

All relevant data are within the paper.
